# Supplementary material for: Economic and econometric methods to measure the illicit tobacco trade: A scoping review
Source: PLOS Glob Public Health. 2026 Mar 23;6(3):e0006118. doi: 10.1371/journal.pgph.0006118 (PMC13008078; doi:10.1371/journal.pgph.0006118)
Supplement: S1 File — Full review protocol describing the objectives, inclusion criteria, search strategy, and methods used for data charting and synthesis, developed in accordance with the Joanna Briggs Institute framework for scoping reviews. (DOCX) [file pgph.0006118.s001.docx]

**Supplementary Information S1: The economic or econometric methods used to measure changes in illicit tobacco use among people who use tobacco: A Scoping Review Protocol.**

Authors: Pyi Pyi Phyo ^1^, Erwann Sbai ^2^, Natalie Walker ^3^, Braden Te Ao ^4^, Chris Bullen ^3^

1. Doctoral Researcher, Faculty of Medical and Health Sciences, University of Auckland
2. Senior Lecturer, Faculty of Business and Economics, University of Auckland
3. Professor, Faculty of Medical and Health Sciences, University of Auckland
4. Senior Lecturer, Faculty of Medical and Health Sciences, University of Auckland

This protocol was developed in June 2023 and revised in July 2023 to incorporate a revised literature search strategy. An additional revision was conducted in May 2024, integrating feedback from peer reviewers.

**Introduction:**

About ten percent of cigarettes in the world are sourced from the ‘black market’ - hereafter referred to as the illicit tobacco market. The World Health Organization (WHO) defines “illicit trade as any practice or conduct prohibited by law and related to production, shipment, receipt, possession, distribution, sale or purchase, including any practice or conduct intended to facilitate such activity” [1]. The illicit tobacco trade undermines public health, government revenue, and control policies [2]. It also threatens countries’ national security by facilitating corruption and undermining governance, linking with terrorist groups, promoting other crimes, and funding additional criminal activities, including money laundering [3].

To combat the illicit tobacco trade, the WHO Protocol to Eliminate Illicit Trade in Tobacco Products was developed in 2012 and entered into force in 2018 [4]. To assess if such protocols and policies are effective, it is necessary to be able to estimate the size of the illicit tobacco trade and do so consistently over time[5]. However, calculating the amount of illicit tobacco trade is difficult due to its unlawful and covert nature. Furthermore, there is evidence that tobacco companies manipulate by providing research funding to produce flawed and inflated estimates of the illicit tobacco trade. A rise in the illicit tobacco trade is a principal argument that the tobacco industry uses to reverse or slow down the implementation of tobacco control policies, such as tobacco taxation, pictorial warnings, and marketing restrictions [6]. Tobacco companies also smuggle their products into the illicit tobacco trade to avoid paying the exercise tax and to protest the regulations against their business [7].

According to the Illicit Tobacco Trade Index, New Zealand (NZ) is among the leading countries in the world combatting illicit tobacco[5]. However, the extent of the illicit trade in NZ is disputed. Estimates for illicit tobacco trade in NZ vary from study to study. For example, a study conducted in 2013-2014 by researchers employed by the non-governmental tobacco control advocacy group ASH (Action on Smoking & Health) found that only 1.8-3.9% of the total national tobacco consumption in 2013 in NZ was illicit [8]. In contrast, a survey by Oxford Economics, funded by the tobacco company Philip Morris, estimated that 2017 more than 10% of cigarettes used in NZ were illicit [9].

The tobacco industry’s estimates of high levels of illicit trade feed into their arguments that stricter tobacco control measures will foster increased illicit trade and crime [10]. However, Gallagher et al. suggested that tobacco control measures are not significant drivers of the illicit trade in NZ. The NZ media reported that the illicit tobacco trade has increased due to high legal cigarette prices from tobacco control measures [11]. Other research has also shown that tobacco control measures (tax and non-tax policies) did not cause an increase in the illicit tobacco trade, in contrast to the claims of the tobacco industry [12].

In 2021, the NZ government published an Action Plan to achieve the Smokefree Aotearoa 2025 goal (Ministry of Health NZ, 2021). The plan outlined strict tobacco control strategies, such as only permitting authorized retailers to sell tobacco products and mandating very low-nicotine-content tobacco. The government then introduced legislation - the Smokefree Environments and Regulated Products (Smoked Tobacco) Amendment Act 2022 (SERPA), which came into force on the 1st January 2023 [13]to accelerate the decline in smoking prevalence in NZ to 5% or below by 2025 ([14]. Furthermore, selling tobacco to anybody born on or after Jan. 1, 2009, would be illegal, according to SERPA. Given such major changes to tobacco control, it was considered that it would be important to estimate the illicit tobacco trade as accurately as possible over time, as these policies came into effect, to detect trends in illicit trade, in particular early signs of increased illicit activity.

During the course of this scoping review, a new coalition government was formed in NZ - one that took a different stance on tobacco control from its predecessor. Just four months after taking office in December 2023, in March 2024, the new government repealed the Smokefree Environments and Regulated Products (Smoked Tobacco) Amendment Act, citing as a contributing rationale concern about a potential surge in illicit tobacco trade [15]. Such policy reversals highlight the need to gather independent evidence of the dynamics of the illicit tobacco trade in NZ.

A range of methods for measuring the size of the illicit trade in any jurisdiction is recommended by expert bodies, including the World Bank, Tobacconomics, the American Cancer Society Toolkit, and the European Union. The methods include surveys examining the cigarette packs used by people who smoke tobacco, discarded packs, interviewing people who smoke about their engagement with the illicit tobacco market, interviews with experts in Customs, consumption gap analysis, and econometric models[16]. No method is sufficient by itself. Rather, it is recommended that multiple methods be used to estimate the correct size of illicit trade in a country (Stoklosa et al., 2020) to enable triangulation of estimates.

In a project measuring illicit trade over the years 2023-2026, which the University of Auckland is conducting for the NZ Ministry of Health, the following methods are being used: discarded pack surveys, interviews with customs officials, social media surveillance, and consumption gap analysis. Consumption gap analysis uses existing data on smoking prevalence and tobacco industry data provided to the Ministry of Health to estimate the difference in the amount of tobacco legally released into the market and the amount consumed. Another approach recommended by international institutions, such as the World Bank and Tobacconomics, involves using more advanced economic and econometric methods[16-18].

**Rationale for a scoping review:**

While the international toolkits and guidelines suggest using economic or econometric methods to measure illicit tobacco trade, the specific methods are not outlined. Economic methods have been applied in tobacco control research for various purposes – to examine relationships among taxation, price, consumption, and disease outcomes; to model nicotine addiction in the context of rational economic behaviour; to analyse how cigarette taxation may shift demand toward other tobacco products; to analyse the role of advertising and its bans in demand for cigarettes; and to examine the influence of information dissemination in consequences of tobacco use, smoke-free laws and net contributions of tobacco to economies [19]. In contrast, econometrics is used to quantify economic phenomena using economic theory, mathematics, and statistical inference [20]. Econometric calculations can be used to estimate how price changes affect demand for illicit tobacco and to explore the relationship between legal sales and variables associated with the demand for tobacco and related to smuggling [21].

**Aim:**

To identify the suitable economic or econometric methods to be used to measure the size and trends in the illicit tobacco trade in NZ. This information is particularly crucial for NZ, given its unique status as a geographically isolated island nation with strong tobacco control policies. Moreover, we aim to consider the uncertainty surrounding the implementation and timelines of tobacco control policy measures and potential impacts on priority population groups from a health equity perspective.

**Research questions:**

The scoping review aims to answer the following questions:

- What economic or econometric methods should be used to measure the size of the illicit tobacco trade?
- What strengths and limitations do these methods have when measuring illicit tobacco use from a health equity perspective?
- Can these methods be used to assess the size and impact of the illicit tobacco market in priority populations (e.g., subpopulations with higher smoking prevalence than the general population, such as Indigenous peoples, and people with low education)?

**Methods:**

The review will follow the scoping review guidance developed by the University of South Australia [22] and the guidance on evidence synthesis developed by the Joanna Briggs Institute[23]. The scoping review will be reported according to the criteria outlined in the PRISMA extension for scoping reviews [24].

**Search Strategy:**

The research question uses the PCC (Population, Concept, Context) framework to identify the following search terms: Population: Active tobacco users; Concept: Economics or Econometric Methods; Context: Globally and in NZ.

Based on the research question, we devised the search strategy outlined in Table A1.

Table A1. Search strategy.

| Concept 1 | Concept 2 | Concept 3 | Concept 4 | Concept 5 |
| --- | --- | --- | --- | --- |
| Illegal | Tobacco products | Econometric/ Economic | Methods | Exclusion |
| illegal OR unlawful OR illicit OR smuggl* OR criminal OR prohibit* OR banned OR contraband | tobacco OR cigarette* OR “roll your own” OR roll-your-own OR “hand rolled cigarette” OR Illicit-Tobacco-Trade | econometric* OR economic* OR tax* OR cost* OR price* OR demand* | model* OR estimat* OR gap-analysis OR experimental-market-place | NOT drugs OR NOT “substance use” OR NOT children OR NOT adolescent* |

We will use the search strategies outlined in Table 1 for the database searches shown in Table A2.

Table A2. Databases and search strategies

| Database | Single or Multiple Search Boxes | Search Strategy | Query Strings |
| --- | --- | --- | --- |
| PubMed | Single | (illegal OR unlawful OR illicit OR smuggl* OR criminal OR prohibit* OR banned OR contraband) AND (tobacco OR cigarette* OR “roll your own” OR roll-your-own OR “hand rolled cigarette” OR Illicit-Tobacco-Trade) AND (econometric* OR economic* OR  tax* OR cost* OR price* OR  demand*) AND (model* OR estimat* OR gap-analysis OR experimental-market-place) NOT (drugs OR “substance use” OR children OR adolescent*) | Abstract  All types of studies  2010-2023 |
| CINAHL | Multiple | (illegal OR unlawful OR illicit OR smuggl* OR criminal OR prohibit* OR banned OR contraband) AND (tobacco OR cigarette* OR “roll your own” OR roll-your-own OR “hand rolled cigarette” OR Illicit-Tobacco-Trade) AND (econometric* OR economic* OR  tax* OR cost* OR price* OR  demand*) AND (model* OR estimat* OR gap-analysis OR experimental-market-place) NOT(drugs OR “substance use” OR children OR adolescent*) | Abstract  All types of studies  2010-2023 |
| EMBASE | Single | (illegal OR unlawful OR illicit OR smuggl* OR criminal OR prohibit* OR banned OR contraband) AND (tobacco OR cigarette* OR “roll your own” OR roll-your-own OR “hand rolled cigarette” OR Illicit-Tobacco-Trade) AND (econometric* OR economic* OR  tax* OR cost* OR price* OR  demand*) AND (model* OR estimat* OR gap-analysis OR experimental-market-place) NOT(drugs OR “substance use” OR children OR adolescent*) | Abstract  All types of studies  2010-2023 |
| EconLit | Multiple | (illegal OR unlawful OR illicit OR smuggl* OR criminal OR prohibit* OR banned OR contraband) AND (tobacco OR cigarette* OR “roll your own” OR roll-your-own OR “hand rolled cigarette” OR Illicit-Tobacco-Trade) AND (econometric* OR economic* OR  tax* OR cost* OR price* OR  demand*) AND (model* OR estimat* OR gap-analysis OR experimental-market-place) NOT(drugs OR “substance use” OR children OR adolescent*) | Optional (Abstract)  All types of studies  2010-2023 |
| ABI/Inform | Multiple | (illegal OR unlawful OR illicit OR smuggl* OR criminal OR prohibit* OR banned OR contraband) AND (tobacco OR cigarette* OR “roll your own” OR roll-your-own OR “hand rolled cigarette” OR Illicit-Tobacco-Trade) AND (econometric* OR economic* OR  tax* OR cost* OR price* OR  demand*) AND (model* OR estimat* OR gap-analysis OR experimental-market-place) NOT(drugs OR “substance use” OR children OR adolescent*) | Anywhere except Full Text  All types of studies from 01-January to 2023 |

A preliminary search found that Scopus produced little relevant papers, and so this database was removed. We will confine our search to PubMed, CINAHL, EMBASE, EconLit, and ABI/Inform.

**Additional Search**

We will also search Medline and the economic working paper platforms: SSRN and IDEAS as outlined in Table A3.

Table A3. A data search in additional databases

| Database | Single or Multiple Search Boxes | Search Strategy | Query Strings |
| --- | --- | --- | --- |
| MEDLINE | Multiple | (illegal OR unlawful OR illicit OR smuggl* OR criminal OR prohibit* OR banned OR contraband) AND (tobacco OR cigarette* OR “roll your own” OR roll-your-own OR “hand rolled cigarette” OR Illicit-Tobacco-Trade) AND (econometric* OR economic* OR  tax* OR cost* OR price* OR  demand*) AND (model* OR estimat* OR gap-analysis OR experimental-market-place) NOT (drugs OR “substance use” OR children OR adolescent*) | Abstract  All types of studies  2010-2023 |
| SSRN | Single Box | (illegal OR unlawful OR illicit OR smuggl* OR criminal OR prohibit* OR banned OR contraband) AND (tobacco OR cigarette* OR “roll your own” OR roll-your-own OR “hand rolled cigarette” OR Illicit-Tobacco-Trade) AND (econometric* OR economic* OR  tax* OR cost* OR price* OR  demand*) AND (model* OR estimat* OR gap-analysis OR experimental-market-place) NOT (drugs OR “substance use” OR children OR adolescent*) | Abstract  All types of studies  2010-2023 |
| IDEAS | Single Box | (illegal OR unlawful OR illicit OR smuggl* OR criminal OR prohibit* OR banned OR contraband) AND (tobacco OR cigarette* OR “roll your own” OR roll-your-own OR “hand rolled cigarette” OR Illicit-Tobacco-Trade) AND (econometric* OR economic* OR  tax* OR cost* OR price* OR  demand*) AND (model* OR estimat* OR gap-analysis OR experimental-market-place) NOT (drugs OR “substance use” OR children OR adolescent*) | Abstract  All types of studies  2010-2023 |

Authors of primary sources or reviews will be contacted for further information, where required. We will also include articles and studies based on tobacco control experts and economists and econometricians’ opinions. Following the methods of the University of Otago ([25], we will search the grey literature on Google, using the following:

- "illicit tobacco" filetype: pdf
- "illicit tobacco" inurl: govt
- related: illicit tobacco. Govt. nz
- "illegal tobacco" filetype: pdf
- "illegal tobacco" inurl: govt
- related: illegal tobacco. Govt. nz

**Eligibility Criteria:**

We will identify the publications that meet all the inclusion criteria for selection.

**Inclusion Criteria:**

- All publication types, including systematic reviews, meta-analyses, rapid reviews, scoping reviews, narrative reviews, qualitative research, simulation modelling studies, reports, and guidelines and reports from international organizations (such as the WHO and the World Bank);
- Publications in English.
- Publications that examine the economic or econometric models/methods in the illicit tobacco trade *;

*Publications that examine the other illicit trade or related topics were included as the research team decided that these methods could also be applied to the illicit tobacco trade.

- Publications from 2010 ** onwards.

** 2010 was selected a cut-off date to capture the most recent methods/models.

**Exclusion Criteria:**

- Publications that do not discuss economic or econometric models/methods
- Publications in languages other than English
- Publications before 2010**

** to capture the most current methods in this field.

(Note: Since this is an examination of the methods, studies’ funding by the tobacco industry is not a criterion for exclusion in the review).

The grey literature published outside the peer-reviewed journals will be included. We will search for grey literature on Google.

**Study Selection Process:**

We will use Rayyan software for the study selection process because it offers a free version, enabling many researchers to participate. Additionally, it provides a blinding mode, meaning researchers do not know which studies others have selected, thus enhancing the rigor of the research [26]. The primary reviewer will remove any duplicate articles/documents identified by Rayyan. During the initial screening phase, identified studies will be selected based only on the title. All the studies that potentially examine the economic or econometric methods of illicit tobacco trade will be selected, regardless of their methods. During the second screening phase, studies will be selected after reviewing the abstract and keywords. The third screening phase will involve reading the full papers. Where there is a lack of consensus between the team for any given paper, a decision will be made after discussion and consensus. The detailed study selection process can be seen in Figure A1.

Import all the research results into Rayyan.

Remove duplicates with Rayyan.

Initial Screening (Title)

Second screening (Abstract and keywords)

Third screening: Full paper

Final number of studies.

Figure A1: Flow chart of the study selection process

**Data Extraction**: We developed a customized data extraction form in Excel ^TM.^ The headings are presented in Table A4.

Table A4: Titles for Data Extraction

| **CGA-related studies** | **Non-CGA-based studies** |
| --- | --- |
| Authors | Authors |
| Year of publication | Year of publication |
| Origin/Country of origin | Origin/Country of origin |
| Aim/Purpose | Aim/Purpose |
| Type of tobacco control policies in the current study context (Existing policy) | Type of tobacco control policies in the current study context (Existing policy) |
| Potential policy scenario (Future policy changes) | Potential policy scenario (Future policy changes) |
| Data analysis and data source | Data collection and data analysis (Models) |
| Unit of measurement | - |
| Comment | Strengths and limitations of models (in a separate table) |

We reviewed assessment checklists such as the Consolidated Health Economic Evaluation Reporting Standards (CHEERS) checklist (Kunst et al., 2023), the National Institute for Health and Care Excellence (NICE) Methodology Guide quality checklist (NICE, 2012) and Huang et al.'s proof-of-concept quality assessment framework for tobacco models. However, these models were unsuitable for assessing our review's strengths and weaknesses. Therefore, based on the checklist by Huang et al. (2023), we developed a simple checklist to evaluate the models for our scoping review (Table A5)[27]. This checklist includes consideration of health equity.

Table A5: Detailed checklist to analyse the strengths and weaknesses of the models including a health equity perspective.

| Population | The model population data represents the population to which the model policies will apply. |
| --- | --- |
| Baseline Scenario | The publication captures the baseline/existing scenario. |
| Policy Scenario | The publication captures the future impact of policy changes. |
| Demand Analysis | The model analysed/forecasted the demand for tobacco based on policy changes. |
| Transparency | Technical or non-technical documents are available to provide model transparency. |
| Equity | The model explored the equity impact of policies. |

**Data Combination, Summarizing, and Reporting**

We will write the scoping review report according to the checklist items in PRISMA-ScR [24]

Charts, tables, or other presentation formats will be used as appropriate. We will discuss the findings from the scoping review and answer the research questions.

References

1. World Health Organization. WHO Framework on Tobacco Control. WHO. 2003: e1001249.

2. Anonymous WHO FCTC Secretariat, director. Protocol to Eliminate Illicit Trade in Tobacco Products; 2019. Geneva: WHO FCTC.

3. Department of State, United States of America. The global illicit trade in tobacco: A threat to national security . 2015.

4. WHO FCTC Secretariat. Protocol to Eliminate Illicit Trade in Tobacco Products
Brochure. 2022.

5. Ulep VG, Lavares MP, Francisco A. Measuring the capacity to combat illicit tobacco trade in 160 countries. Globalization and health. 2021;17: 130–8. doi: 10.1186/s12992-021-00783-4.

6. WHOFCTC. The Tobacco Industry and
the Illicit Trade in Tobacco Products. WHO. 2021.

7. Evans-Reeves K, Rowell A. Tobacco industry rallies against illicit trade – but have we forgotten its complicity? . 2025.

8. Ajmal A, U VI. Tobacco tax and the illicit trade in tobacco products in New Zealand. Australian and New Zealand Journal of Public Health. 2015;39: 116–120. doi: 10.1111/1753-6405.12389.

9. Oxford Economics. Asia Illicit Tobacco Indicator 2017: New Zealand. 2018.

10. University of Bath. Tobacco Tactics: Illicit Tobacco Trade . University of Bath. 2021. Available: <https://tobaccotactics.org/article/illicit-tobacco-trade/>.

11. Gallagher A, Robertson L, Hoek J, Wilson N, Edwards R. Illicit tobacco trade and the Smokefree Aotearoa 2025 Goal: Arguments and evidence. 2021.

12. Paraje G, Stoklosa M, Blecher E. Illicit trade in tobacco products: recent trends and coming challenges. Tob Control. 2022;31: 257–262. doi: 10.1136/tobaccocontrol-2021-056557.

13. Parliamentary Counsel Office. Smokefree Environments and Regulated Products (Smoked Tobacco) Amendment Act 2022. <https://www.legislation.govt.nz/act/public/2022/0079/latest/whole.html.> 2022.

14. Ministry of Health New Zealand. History of Smokefree Aotearoa 2025. Ministry of Health, New Zealand. 2023. Available: <https://www.health.govt.nz/our-work/preventative-health-wellness/smokefree-2025/smokefree-aotearoa-2025-action-plan/smokefree-aotearoa-2025-action-plan/history-smokefree-aotearoa-2025>.

15. Edwards R, Bullen C, Hoek J, Tukuitonga C, Waa A, Walker N. Public health vandalism: new Government scraps world-leading smokefree legislation. New Zealand Medical Journal. 2023;136.

16. Ross H. Understanding and Measuring Cigarette Tax Avoidance and Evasion : A Methodological Guide : Tobacconomics, Health Policy Center, Institute for Health Research and Policy, University of Illinois at Chicago; 2015.

17. Merriman D, Yurekli A. Economics of Tobacco Toolkit, Tool 7 : Understand, Measure, and Combat Tobacco Smuggling: World Bank, Washington, DC; 2013.

18. Stoklosa M, Paraje G, Blecher E. A Toolkit on Measuring Illicit Trade in
Tobacco Products. A Tobacconomics and American Cancer Society Toolkit. Chicago: Tobacconomics, Health Policy Center, Institute for Health Research and Policy, University of Illinois at Chicago; 2020.

19. Warner KE, Chaloupka FJ. The Economics of Smoking. NBER Working Paper Series. 1999: 7047. doi: 10.3386/w7047.

20. Ouliaris S. What is Econometrics? Taking a theory and quantifying it. 2011.

21. [Anonymous]. Economics of Tobacco Toolkit, Tool 7 : Understand, Measure, and Combat Tobacco Smuggling. Washington (DC): World Bank; 2013.

22. University of South Australia. Overview of scoping review. University of South Australia. 2023. Available: <https://guides.library.unisa.edu.au/ScopingReviews/ScopingReviewOverview>.

23. Micah P, Godfrey C, McInerney P, Munn Z, Tricco AC, Khalil H. Chapter 11: scoping reviews (2020 version). <https://jbi-global-wiki.refined.site/space/MANUAL/4687342/Chapter+11%3A+Scoping+reviews.> 2020.

24. Tricco AC, Lillie E, Zarin W, O'Brien KK, Colquhoun H, Levac D, et al. PRISMA Extension for Scoping Reviews (PRISMA-ScR) : Checklist and Explanation. 2018.

25. University of Otago. Using Google to Get Started. Wellington Medical and Health Sciences: Library Guides. 2023. Available: <https://otago-med.libguides.com/greylit/google>.

26. Rayyan. Rayyan. Rayyan. 2022. Available: <https://www.rayyan.ai/>.

27. Huang V, Head A, Hyseni L, O'Flaherty M, Buchan I, Capewell S, et al. Identifying best modelling practices for tobacco control policy simulations: a systematic review and a novel quality assessment framework. Tob Control. 2023;32: 589–598. doi: 10.1136/tobaccocontrol-2021-056825.
