# Supplementary material for: Economic and econometric methods to measure the illicit tobacco trade: A scoping review
Source: PLOS Glob Public Health. 2026 Mar 23;6(3):e0006118. doi: 10.1371/journal.pgph.0006118 (PMC13008078; doi:10.1371/journal.pgph.0006118)
Supplement: Attachment — Submitted filename: Responses to the comments.docx [file pgph.0006118.s004.docx]

Addressing the comments of the editor and reviewers

Thank you for the helpful comments and feedback; we have addressed them as follows:

| Editor’s and reviewers’ comment | Address | Page |
| --- | --- | --- |
| Editor’s comments | | |
| I noticed that the opposite of inclusion are written as exclusion criteria. Could you remove exclusion criteria (these need not necessary to be there). | Deleted the exclusion criteria | #6 |
| If you had to exclude any articles among those included after title, abstract or full text review, please state the reasons. | The reasons for exclusion were added. | #6 and #7 |
| **Reviewer 1 comment** | | |
| Authors should mind about the grammar and use of tenses when reporting in the manuscript | Thank you for the feedback, we have carefully reviewed (and edited) the text to address this point. |  |
| **Abstract**  In the method section, the statement “Google Scholar for grey literature”, these two mean different things and therefore the authors need to separate them and not one leading to the other  The authors used Google scholar only as a database, were there no other data bases used such as PubMed, Medline and many others. Didn’t this create publication selection bias and how was this overcome? | The ‘“Google Scholar for grey literature” text has been removed.  We used Google following the University of Otago’s method;  We searched six databases (  PubMed, CINAHL, EMBASE, EconLit, and ABI/Inform and MEDLine) and two economic working paper platforms (SSRN and IDEAS). These are key databases for public health and economics. Due to the limitations noted in the abstract, we could not add the details.  We have modified the text: “We searched six key databases for public health and economic papers (PubMed, CINAHL, EMBASE, EconLit, and ABI/Inform and Medline) and two economic working paper platforms (SSRN and IDEAS) for papers. Additionally, we searched for grey literature on Google and added the expert-selected articles.“ | #2 |
| Authors used publications in English, were there good relevant publications in other languages which were excluded? Again how was this overcome to avoid language bias? | We extensively searched the key public health and economics databases and added expert-suggested articles and grey literature. By doing this, we are confident there is minimal selection bias. We have discussed the details of this in the discussion section of the main article. | #70 and #71 |
| In the results section, the statement “23 used other economic or econometric models to assess tobacco or marijuana demand”. What were those other economic or econometric models? | We have added the models in the results section of the abstract as follows:  “The review included 39 studies: 16 applied Consumption GAP Analysis (CGA), and 23 used other economic or econometric models such as Hursh And Silberberg's Exponentiated Model, Discrete Choice Models, Extended Cost–Benefit Analysis and A Static Partial Equilibrium Model, Consumption Models, Consumption Models, Risk Prediction Models, A Forward-looking Behavioural Model, Integrated Micro-Macro Model of Demand, Endogenous Switching Regression Model, Multiple and Non-Linear Regression Model, Prevalence Projection Model, Demand-driven Analytical Model, Econometric Regressions Model, Econometric Modelling and two way fixed effects model. Only six of the 39 studies addressed health equity. “ | #2 |
| In the results section “marijuana” is mentioned and yet it was not in the title and aim of the review | We only aimed to look at the illicit tobacco trade.  “Marijuana” is mentioned because of the selection criteria, which state “We included English publications from 2010 onwards that examined economic or econometric models assessing changes in illicit tobacco trade or related topics.”  We have clarified this in the discussion section of the main article. | #2, #70, #71 |
| There are no results for the “trends” in the abstract, as mentioned in the conclusion | This was inadvertently omitted but has been added now.  “CGA was used to estimate the existing illicit tobacco market size and assess the market trends over the past years, while the other models were used to assess and quantify the existing behavior and potential behaviors of engaging with different tobacco and other products, including illicit tobacco.”  In the conclusion section, we added  “Measuring the illicit tobacco trade is challenging due to its covert nature, methodological constraints, and limited high-quality data. Economic method or econometric method selection depends on the research objective: CGA is suitable for assessing national market trends but is limited in evaluating subpopulations or future policy impacts. Economic models are suitable for understanding or predicting user behaviour, including from a health equity perspective.” | #2 and #3 |
| **Introduction**  The introduction seems to be well written except a few issues including: On the illicit tobacco trade, what are the various tobacco products that are traded and does this include the products that are home grown? | To address the point raised we have added: “According to data collected from NZ Customs, many different types of illicit tobacco were seized during this period – illicit cigarettes, illicit loose tobacco imported from other countries (especially Tonga), chewing tobacco, water pipe tobacco, and domestically grown and produced loose tobacco (Bullen et al., 2023). Under the NZ Customs and Excise Act 2018, adults are permitted to cultivate up to 5 kg of tobacco annually for personal use but the sale or distribution of home-grown tobacco is prohibited and illegal (New Zealand Customs Service, 2023)“ | #4 |
| **Methods:**  Suggest it is changed to “Methodology” | We have changed the heading to “Methodology” | #6 |
| Line 2 in the methods, grammar issue that needs to be corrected. Authors should use past tense | Thank you for pointing this out. We have edited the text accordingly. | #6 |
| The inclusion criteria “Publications that examine the other illicit trade or related topics were included..” I think these should not be included since they are out of scope. The implication of the findings can just be used in other relevant areas and to form a basis to do the same study focusing on those other areas. | Thank you for the comment. We considered this point when we developed the protocol. However, we kept the criteria more open because we wanted to explore the economic and econometric methods as much as possible. Although our search strategy focused on tobacco, we included studies that examined illicit tobacco or other tobacco products or other illicit products like marijuana.  We have addressed this point in the discussion. | #70 #71 |
| The authors have not mentioned dissertations or theses that form the gray literature, couldn’t these also be useful. Isn’t there a publication bias? | We added all the publications, including theses or dissertations, if they met the inclusion criteria. We have modified the text of the eligibility criteria accordingly. | #6 and #7 |
| In the search strategy, PubMed and other databases were used but in the abstract only Google scholar is mentioned. Authors need to clear this! | We mentioned that we searched six databases, plus Google for grey literature. Full details were not added given word count limitations.  However, we have now added to the abstract all the databases we searched. There was a typo error as we searched for grey literature on Google not on Google Scholar. | #8 |
| The keywords that were used in the search are missing, and how they were combined. See the Boolean operators in the search strategy | The overall search strategy, as well as details for each database, were listed in the protocol; we have added the overall search strategy in the main manuscript as Table 1. | #7 and #8 |
| The disagreement on which papers should be incorporated in the study, discussion among the 3 authors’ reviewers and agreement, was this the only based choice to go over it? | Our discussion is based on the criteria, and the key criteria is the economic or econometric methods.  We have modified the text accordingly to make this clearer. | #8 |
| How was the synthesized data summarized? | The format to synthesize the data was detailed in the protocol. We have now added this information to the main manuscript as Table 2. | #9 |
| **Results**  In the results section, were there regional distributions of the papers? | Yes - we have added this information to the paper. | #10 |
| And what was the contribution of those papers such as original papers, reviews, reports, dissertation/theses etc. | We have added this information to the manuscript. | #10 |
| When the” an ‘uplift factor” was used, there any differences in the outcomes when compared to the original model? | We have added this information to the manuscript. | #11 |
| CGA were excluded but the authors have not mentioned the other models that were used i.e. what are those Economic/econometric Models that were used for Critical Appraisal? What are their strength and drawbacks? | We have added the models used into the critical appraisal; our approach was to appraise from the health equity perspective of each study in Table 6. | #62 |
| Not all the research questions have been addressed i.e. where is the results for the trend and health equity? | Thank you, assessing the trend is not part of our research session but our findings from CGA as we went through the papers and methods. From the methods, we found out that CGA is useful for assessing the trends from estimating the illicit tobacco in the past, while other economic/econometric models could be applied to estimate the past or current illicit tobacco use behaviour or predict the future. We have assessed all the models from health equity perspectives. We have revised this in the results and in discussion sessions. | #10,11,12 #30  #62 to #71 |
| **Conclusion** The authors should have conclusions about all the research questions raised. | We have added this information to the manuscript. | #71 and #72 |
| **Reviewer 2** |  |  |
| The manuscript presents a scoping review of economic and econometric methods to measure the illicit tobacco trade.  The reviewed promises a lot and I was a little bit disappointed with the lack of depth in the findings.  The manuscript contains several typographical errors so would benefit from careful proofreading. | Thank you for your review and comments.  We have summarised the models and main findings in the main paper. Two tables were placed into Appendix 2 and now we incorporated them into the main paper.  We have carefully proof-read the paper. |  |
| **Background**  Suggest breaking this down into more than one paragraph to enhance readability. | We have broken the section into Additional paragraphs, as suggested. | #4 and #5 |
| For the benefit of less specialist readers, I suggest saying a bit more about what is meant by economic/econometric methods / how these are defined, and justify the focus of the review on these over the other approaches. Suggest explaining more about what the CGA method is. | These definitions were explained in the study protocol and attached in an appendix. We have now added them to the introduction. | #4 #5 |
| **Methods** OSF – should be ‘Open Science Framework’. | Thank you. We have made this change. | #6 |
| * This point could do with more explanation – what sort of other illicit trade? Why was this deemed applicable to tobacco? Also, was the search designed to capture all relevant papers on other types of illicit trade? | We have added the detail requested | #6,#7 and #8 |
| The presentation of the eligibility criteria is a bit hard to follow with the multiple asterisks and final point in brackets. Could be revised for readability. | We have revised the text accordingly. | #6 and #7 |
| Data extraction: please provide details on what information was extracted from the papers. The lack of detail here is ultimately reflected in the findings – see my comment on this below. | This information was in protocol, and appendices. We have now added the detail into the main manuscript. | #9 |
| **Results** I found the results quite disappointing, in that they did not really address the main research question ‘What economic or econometric methods should be used to measure the size of the illicit tobacco trade?’ The findings were very descriptive, focussing more on what methods have been used, rather than the advantages and disadvantages of the different approaches. Given that this is a scoping review, I think the findings could have been more discursive, drawing on what the authors of those papers identified as strengths and weaknesses as well as identifying the methods themselves. | We have made major revisions to the results section to address this comment. We have added a summary table of the models and objectives with strengths and limitations. | #10 to #67 |
| One of your research questions is around suitability of methods for assessing equity, but you say CGA cannot be used to investigate this - it is a bit unclear why this approach was nonetheless included in the review. | We have added the CGA to the equity assessment. | #62 |
| A summary table would be helpful, setting out the types of methods, key points about how they work, main strengths and weaknesses etc. | We have now done this | #30 to #35 |
| **Discussion** The paragraph where you talk about the Likert scale was not clear | We have revised the paragraph accordingly | #67 to #71 |
| You conclude by saying that triangulation is important – it would be helpful to provide some more concrete recommendations, e.g. by bringing some of the other approaches back into the discussion – surveys etc – and talking about what this triangulation should/could involve. | We have revised the conclusion section accordingly. | #71, #71 and #72 |
