# Supplementary material for: Economic and econometric methods to measure the illicit tobacco trade: A scoping review
Source: PLOS Glob Public Health. 2026 Mar 23;6(3):e0006118. doi: 10.1371/journal.pgph.0006118 (PMC13008078; doi:10.1371/journal.pgph.0006118)
Supplement: Attachment — Submitted filename: Response to Reviewers Comments.docx [file pgph.0006118.s005.docx]

Responses to the Editor and Reviewers' Comments

| 1. Reviewer 3 Feedback | Study should be aligned with the objectives, and deviation of objectives to satisfy the data requirement can not be justified. As the objective and search strategy is for tobacco, discussion on other substances is causing as serious bias. | We appreciate the reviewer’s comment. As this is an innovative study, we intentionally adopted broader inclusion criteria to capture widely available methods across illicit trade, not limited to tobacco. We were transparent in our methodology and findings, ensuring that this approach did not introduce serious bias, and it remains consistent with our stated objectives. |
| --- | --- | --- |
| 2. Reviewer 4 Feedback | The scoping review is methodologically sound and rich in content, but it would benefit from transparency in data methods and appraisal. The limitations need to be highlighted clearly and make future recommendations based on the same.  Kindly report both abstract and manuscript using the PRISMA-ScR checklist. In the methods section of the abstract, include quality assessment. | Thank you for your feedback.  In the abstract, we added:  “We examined the methods, assessing their strengths and limitations from a health equity perspective, and evaluated their applicability to priority populations (rather than assessing the quality of individual models)”  In the main manuscript , We have added (see page 9, paragraph 1):  “We examined the strengths and limitations of these methods through a health equity lens, emphasising their relevance to priority populations rather than the quality of individual models.” |
|  | The review explicitly states that "no assessment was undertaken of the individual models employed" and that CGA studies were excluded from critical appraisal because the method is based on mathematical calculations and irrelevant to equity appraisal criteria. Thus, it can be mentioned clearly in limitation and applied in future recommendation. | Thank you, we have modified the method session as follows: (see page 9, paragraph 1)  “Studies that used CGA were included as a single category in the critical appraisal, as they utilised the same methodological approach.”  In the Discussion Section, we have added (see Page 44, Paragraph 2)  “The CGA method has been utilised to estimate both the current extent of illicit trade and historical trends, depending on the availability of data. However, the method has several limitations. It cannot be used to estimate future illicit tobacco trade, and its estimates are calculated only at the national level. These limitations mean that data for specific population subgroups are not available, which limits the method's applicability in considering health equity. One possible reason for this limitation is the nature of the demand and supply data, which are typically available only at the national level. |
|  | The review highlights that "only six of the 39 studies addressed health equity" and explicitly notes that CGA studies "did not reflect equity aspects" because it typically assesses illicit trade at the national level and lacks disaggregated data for subpopulations. Thus, review should also offer more concrete suggestions on how economic models could better integrate health equity components where currently lacking, beyond just stating their suitability. | In the manuscript discussion sessions, we have added:  (see page 47, paragraph3)  “Overall, this review highlights that most economic models used to assess illicit tobacco trade have limited capacity to capture equity dimensions. To strengthen future applications, the studies that applied economic or econometric models should incorporate disaggregated datasets, subgroup analyses, and equity-weighted parameters to better reflect the distributional impacts of tobacco control policies across priority populations. Integrating these approaches would enhance the capacity of existing models to assess not only overall effectiveness but also the fairness and inclusiveness of policy outcomes, thereby advancing a health equity perspective in tobacco control research.” |
|  | The inclusion criteria allow for studies on "other forms of illicit trade (e.g., illicit alcohol, illegal drugs, or various types of nicotine and tobacco products)". However, the population in the PCC framework is restricted to active tobacco users. This is also reflected in concept 5 of exclusion. While justified by the research team for exploring methods, the discussion does not delve into whether these methods are equally applicable or require significant adaptation for tobacco, or if these studies truly enriched the understanding of illicit tobacco trade beyond just methodological exploration. | We have added the following in the discussion (see page 47, paragraph 4).  “The inclusion criteria for the review enabled identification of studies examining other forms of illicit trade, such as illicit alcohol or drugs, while the PCC framework focused specifically on active tobacco users. The use of broader inclusion criteria was intentional and enabled the identification of methodological approaches that could be transferable to illicit tobacco research. However, because the primary focus of this review was illicit tobacco, the search strategy excluded studies centred solely on illicit drugs. Although these additional studies primarily contributed to methodological insights rather than direct evidence on illicit tobacco markets, they were valuable in identifying adaptable analytical techniques—such as behavioural and econometric models—that could be refined and applied within illicit tobacco contexts.” |
|  | The exclusion of non-English language papers due to resource limitations is noted. The review could propose strategies for future reviews to mitigate language bias, such as collaboration with multilingual researchers, or a more targeted search for seminal works in specific non-English languages if resources allow. | Thank you for the comment.  We have added the following text (see page 48, paragraph 2)  “Future reviews could address language bias through multilingual collaboration or targeted searches for key non-English studies. ” |
| Editor Feedback | Please send a completed 'Competing Interests' statement, including any COIs declared by your co-authors. If you have no competing interests to declare, please state “The authors have declared that no competing interests exist”. Otherwise, please declare all competing interests beginning with the statement “I have read the journal's policy and the authors of this manuscript have the following competing interests:” | Thank you, we have added the requested statement to the manuscript. |
|  | Please provide a complete Data Availability Statement in the submission form, ensuring you include all necessary access information or a reason for why you are unable to make your data freely accessible. If your research concerns only data provided within your submission, please write “All data are in the manuscript and/or supporting information files” as your Data Availability Statement. | Thank you, we have added a Data Availability Statement in the submission form. |
|  | Please provide separate figure files in .tif or .eps format only and ensure that all files are under our size limit of 10MB. | Thank you, we have added the files to the submission. |
|  | Please include a separate legend or caption for Figure 1 in your manuscript. | Thank you, we have added the caption for Figure 1.  Figure 1: Flow chart for the study selection using the PRISMA flow diagram (Page et al., 2021). |
|  | We have noticed that you have uploaded Supporting Information files, but you have not included a list of legends. Please add a full list of legends for your Supporting Information files before or after the references list. | Thank you, we have added a full list of legends before the reference list. |
|  | If the reviewer comments include a recommendation to cite specific previously published works, please review and evaluate these publications to determine whether they are relevant and should be cited. There is no requirement to cite these works unless the editor has indicated otherwise. | Thank you. This is not applicable. |
