# Supplementary material for: Economic and econometric methods to measure the illicit tobacco trade: A scoping review
Source: PLOS Glob Public Health. 2026 Mar 23;6(3):e0006118. doi: 10.1371/journal.pgph.0006118 (PMC13008078; doi:10.1371/journal.pgph.0006118)
Supplement: Attachment — Submitted filename: Response to reviewer_06FEB2026.docx [file pgph.0006118.s006.docx]

Response to reviewer

| Comment | Response |
| --- | --- |
| Introduction  Page 5: In the statement “Economic methods have been used in tobacco control research for a variety of purposes: to study the relationships among taxation, price, consumption, and health outcomes; to model nicotine addiction within the framework of rational economic behaviour; to assess how cigarette taxation might shift demand toward other tobacco products; to evaluate the impact of advertising and advertising bans on cigarette demand; and to explore the effects of information dissemination, smoke-free laws, and the overall economic contributions of tobacco (14)”, I suggest changing the phrase “the overall economic contributions of tobacco” to “the economic costs of tobacco”. | Thank you, we have changed the texts to  “the economic costs of tobacco”. |
| Page 5: The statement specific to NZ “This focus was particularly relevant in the NZ context, where the prevalence of tobacco use varies significantly among different ethnic groups and socio-economic strata” can be made more general for nations where tobacco use and related harms disproportionately affect certain population sub-groups including socioeconomically disadvantaged and underserved communities. | We have changed it to “This focus was particularly relevant in the NZ context and other similar contexts, where the prevalence of tobacco use varies significantly among different ethnic groups and socio-economic strata. “ |
| Methodology  Page 8: In Table 2, CGA-related studies are separated from studies using economic or econometric methods. Do the authors consider that CGA-related studies are not economic studies? Consumption gap analysis involves valuation of the size of legal and illegal markets based on consumer demand for the products, which are very well subject of economic research. I would feel more comfortable to name all studies as using economic and econometric methods and divide them into two groups: CGA and non-CGA based studies. | We have changed the headings to  CGA-related studies and non-CGA based studies. |
| We examined the strengths and limitations of these methods through a health equity lens, emphasising their relevance to priority populations rather than the quality of individual models.”—Who are included in the priority populations groups? A clear definition in this section would help readers better understand the relevance of the review to health equity perspective. | We have modified it to  “Priority populations included, but were not limited to, analyses focusing on specific ethnic groups, gender, socioeconomic status, and mental or physical health characteristics associated with increased vulnerability to tobacco use.” |
| The PRISMA diagram in Figure 1 is blurry and can hardly be read. | Thank you , we have saved it as pdf vector and reattached it in the submission. |
| Results  Page 10: “In one study (40)researchers utilised data from the ‘Euromonitor International’, as country-level data were unavailable(40) .” Is there any information in the study reviewed on how Euromonitor International estimate illicit market size? | Yes, the study explained its view on Euromonitor International's illicit tobacco estimate. We have added an additional sentence:  “The study acknowledged that Euromonitor International’s illicit trade estimates were inconsistent; however, it relied on Euromonitor International data on registered sales for certain countries, assuming that there are fewer incentives to manipulate data on tobacco production or legal sales.” |
| Table 3: The column titled “Currency of calculations” makes one to expect that the market value of illicit tobacco is presented in the study which is true for some studies but not all. It is better reflected if you call it “unit of measurement”. Since industry-funded studies were not excluded from the review, I would recommend reporting in a separate column the funder of the study to enable readers to identify industry-funded studies. It is also important to explain that industry-funded studies are prone to overestimate the size of illicit trade. | We have changed it to the “unit of measurement”.  We have added a column to Table 3 indicating whether each study is industry-funded or not. |
| Discussion  Page 44: The limitation that the CGA estimates are calculated only at the national level is not exactly true. It is possible to apply the CGA method to subnational level to obtain estimates for specific geographic areas. See for example method of estimating illicit market size of commercial cannabis in multiple states in USA in: Nigar Nargis, Samuel Asare, J Lee Westmaas, Measuring commercial cannabis availability: findings from a multi-state surveillance study in the US, Health Affairs Scholar, Volume 3, Issue 12, December 2025, qxaf209, <https://doi.org/10.1093/haschl/qxaf209> | We have changed the texts to “It cannot be used to estimate future illicit tobacco trade. Although the method can generate state- or subpopulation-level estimates when sufficient data exist, limited data availability has resulted in its predominant use at the national level. “ |
| The review found studies on illicit tobacco trade mostly do not cover the healthy equity perspective. From Table 6, it looks like a few studies (e.g., Tucker, 2017; Tangtammaruk, 2017, etc.) did have equity perspective. There is need for expanding the discussion on how these studies implemented the equity perspective and what lessons can be drawn from these studies for other approaches to studying illicit tobacco trade. | We have added “ Overall, this review highlights that most economic models used to assess illicit tobacco trade have applied limited equity dimensions. The six studies that included subpopulation analyses conducted limited subgroup analyses based on selected sociodemographic and smoking-related characteristics, including age, sex, ethnicity, income, and smoking status. To strengthen future applications, the studies that applied economic or econometric models should incorporate disaggregated datasets, subgroup analyses, and equity-weighted parameters to better reflect the distributional impacts of tobacco control policies across vulnerable or priority populations. Integrating these approaches would enhance the capacity of existing models to assess not only overall effectiveness but also the fairness and inclusiveness of policy outcomes, thereby advancing a health equity perspective in tobacco control research.” on Page 48  We have also added which subpopulation analysis the study did in Table 6. |
