# Supplementary material for: Economic and econometric methods to measure the illicit tobacco trade: A scoping review
Source: PLOS Glob Public Health. 2026 Mar 23;6(3):e0006118. doi: 10.1371/journal.pgph.0006118 (PMC13008078; doi:10.1371/journal.pgph.0006118)
Supplement: Attachment — Submitted filename: Response to reviewer.docx [file pgph.0006118.s007.docx]

Response to reviewer/Editor

| Journal Requirements:  If the reviewer comments include a recommendation to cite specific previously published works, please review and evaluate these publications to determine whether they are relevant and should be cited. There is no requirement to cite these works unless the editor has indicated otherwise. | There was no such comment. |
| --- | --- |
| Please review your reference list to ensure that it is complete and correct. If you have cited papers that have been retracted, please include the rationale for doing so in the manuscript text, or remove these references and replace them with relevant current references. Any changes to the reference list should be mentioned in the rebuttal letter that accompanies your revised manuscript. If you need to cite a retracted article, indicate the article’s retracted status in the References list and also include a citation and full reference for the retraction notice. | There is no retracted article. However, there are additional references in the Background section that we revised. |
| Dear Authorship team,  Thank you for all the effort in revising this paper multiple times. I know it is time consuming and takes effort. The paper reads well and all reviewer comments have been adequately addressed. I have one minor suggestion to bring this paper methodologically on par with other reviews: this is to include a inclusion/exclusion criteria table. This will be a very quick revision given that you have inclusion criteria is outlined already. If you would like an example, you can look at this paper: <https://academic.oup.com/heapro/article/39/6/daae155/7906019> | Thank you for your review and feedback.  Yes, we have added a table of inclusion/exclusion criteria as you suggested, and the article reads better. |
| Once you address that, I can proceed to accepting your paper. Thanks for the work which will help advance tobacco control efforts and inform policy. | Thank you. |
| Figure Resubmissions:  While revising your submission, we strongly recommend that you use PLOS’s NAAS tool (<https://ngplosjournals.pagemajik.ai/artanalysis>) to test your figure files. NAAS can convert your figure files to the TIFF file type and meet basic requirements (such as print size, resolution), or provide you with a report on issues that do not meet our requirements and that NAAS cannot fix.  After uploading your figures to PLOS’s NAAS tool - <https://ngplosjournals.pagemajik.ai/artanalysis>, NAAS will process the files provided and display the results in the "Uploaded Files" section of the page as the processing is complete. If the uploaded figures meet our requirements (or NAAS is able to fix the files to meet our requirements), the figure will be marked as "fixed" above. If NAAS is unable to fix the files, a red "failed" label will appear above. When NAAS has confirmed that the figure files meet our requirements, please download the file via the download option, and include these NAAS processed figure files when submitting your revised manuscript. | Yes, we have used the tool to change our figure to TIF file type. |
